# Phagocytosis of Necrotic Debris at Sites of Injury and Inflammation

**DOI:** 10.3389/fimmu.2019.03030

**Published:** 2020-01-09

**Authors:** Johannes Westman, Sergio Grinstein, Pedro Elias Marques

**Affiliations:** ^1^Program in Cell Biology, Hospital for Sick Children, Toronto, ON, Canada; ^2^Department of Biochemistry, University of Toronto, Toronto, ON, Canada; ^3^Keenan Research Centre of the Li Ka Shing Knowledge Institute, St. Michael's Hospital, Toronto, ON, Canada; ^4^Laboratory of Molecular Immunology, Department of Microbiology, Immunology and Transplantation, Rega Institute for Medical Research, KU Leuven, Leuven, Belgium

**Keywords:** cell death, necrosis, apoptosis, phagocytosis, inflammation, cell debris, “find-me”, “eat-me”

## Abstract

Clearance of cellular debris is required to maintain the homeostasis of multicellular organisms. It is intrinsic to processes such as tissue growth and remodeling, regeneration and resolution of injury and inflammation. Most of the removal of effete and damaged cells is performed by macrophages and neutrophils through phagocytosis, a complex phenomenon involving ingestion and degradation of the disposable particles. The study of the clearance of cellular debris has been strongly biased toward the removal of apoptotic bodies; as a result, the mechanisms underlying the removal of necrotic cells have remained relatively unexplored. Here, we will review the incipient but growing knowledge of the phagocytosis of necrotic debris, from their recognition and engagement to their internalization and disposal. Critical insights into these events were gained recently through the development of new *in vitro* and *in vivo* models, along with advances in live-cell and intravital microscopy. This review addresses the classes of “find-me” and “eat-me” signals presented by necrotic cells and their cognate receptors in phagocytes, which in most cases differ from the extensively characterized counterparts in apoptotic cell engulfment. The roles of damage-associated molecular patterns, chemokines, lipid mediators, and complement components in recruiting and activating phagocytes are reviewed. Lastly, the physiological importance of necrotic cell removal is emphasized, highlighting the key role of impaired debris clearance in autoimmunity.

## Introduction

Cell death is inherent to living multicellular organisms. It is a key regulator of homeostasis, being required during development, growth and maintenance of tissues; it is also a turning point in the immune response. Healthy humans lose *billions* of cells per day constitutively via the process of apoptotic cell death. Apoptosis, the prototypical form of programmed cell death, was described morphologically in the early seventies (1) as involving cell shrinkage and chromatin condensation, followed by fragmentation of the entire cell into smaller, sealed apoptotic bodies. These apoptotic bodies are promptly cleared by neighboring phagocytes and parenchymal cells through phagocytosis, in this case termed efferocytosis (meaning “carrying to the grave”), without initiating an inflammatory response or disturbing tissue homeostasis.

While apoptosis has been studied most extensively, there are many other ways for cells to experience death. The intrinsic activity of organisms often puts them in contact with extreme temperatures, strong mechanical forces and harmful chemical agents. These situations frequently culminate in a catastrophic form of cell death with loss of plasma membrane integrity and pro-inflammatory properties named necrosis (2). Necrotic cell death can either be accidental or programmed (e.g., pyroptosis and necroptosis), leading to the release of intracellular contents into the extracellular environment. Necrosis differs qualitatively from apoptosis, which is clearly demonstrated by the lack of conversion of necrotic cells into apoptotic bodies, a process that requires enzymatic activity and energy. Importantly, these differences also predict that the means of clearance of the cell debris generated by necrosis vs. apoptosis may be drastically different.

**Figure 1:**
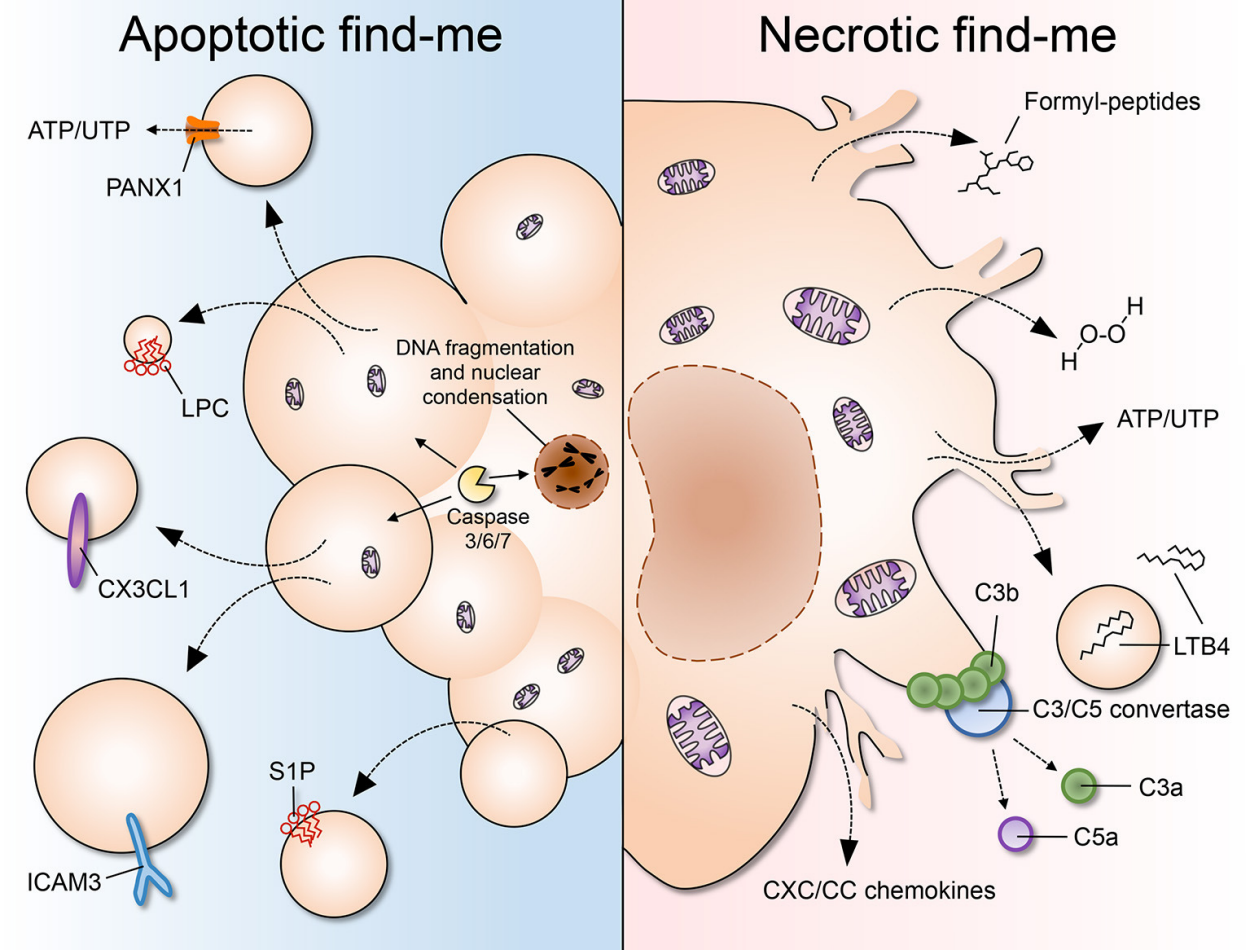
A comparison of apoptotic and necrotic “find-me” signals. **(Left)** Apoptosis is characterized by cell shrinkage, membrane blebbing, DNA fragmentation and nuclear condensation. As cells undergo apoptosis, “find-me” signals such as lysophoshatidylcholine (LPC), CX3CL1, ICAM3, and sphingosine 1-phosphate (S1P) are secreted, exposed on the outer leaflet of the plasma membrane, and/or released via apoptotic bodies or exosomes. Pannexin 1 (PANX1) is an important membrane channel involved in formation of membrane protrusions and ATP/UTP release during apoptosis. LPC, lysophosphatidylcholine; S1P, sphingosine-1-phosphate. **(Right)** Necrosis is considered to be an uncontrolled form of cell death characterized by nuclear and organellar swelling, plasma membrane rupture and leakage of intracellular contents, which many fall into the category of damage-associated molecular patterns (DAMPs or danger signals). “Find-me” signals released by necrotic cells include mitochondria-derived formylated peptides, as well as molecules released from the cytosol such as H_2_O_2_, ATP/UTP, leukotriene B4 (LTB4), and CXC/CC chemokines. LTB4 can also be released via sealed extracellular vesicles. The chemotactic complement components C3a and C5a are generated after complement activation on the surface of necrotic cells.

Efferocytosis has received a great deal of attention in the past decades, and is by now a well-understood process involving dozens of described receptors and molecular effectors (Figure 1). Because of the profusion of studies, a casual reader may be left with the mistaken impression that efferocytosis is the only means of clearance of cell debris in the body. This is certainly not the case, as is most graphically shown by the existence of apoptosis-defective organisms, such as mice deficient in the initiator caspases 2 (3) and 9 (4), and effector caspases 3 (5), 6 (6), and 7 (7), that nevertheless develop and survive rather normally! Clearly, other mechanisms of cell death and debris clearance must exist. The main purpose of this chapter is to review the clearance of cell debris of necrotic origin. Parallels will be drawn between apoptosis and necrosis, stressing how each mode of cell death may produce different “find-me” and “eat-me” signals that will ultimately lead to clearance of debris by different cell types and phagocytic receptors. In addition, the immunological consequences of defective clearance of cell debris will be discussed: this can take the form of delayed tissue regeneration upon injury or even severe autoimmunity in the long-term. In collating the available information on necrotic cell clearance, this review aims to shed new light on diseases in which necrotic debris are central, such as in atherosclerosis, liver injury, arthritis, severe trauma, lupus, and many others.

## Apoptosis and Efferocytosis

Approximately 200 billion cells undergo turnover (ostensibly by apoptosis) every day in the human body (8). Yet, few apoptotic cells are found in the steady state in healthy humans, suggesting that these cells are rapidly cleared. In order to orchestrate efferocytosis, three main signaling programs are required. First, chemotactic “find-me” signals are produced to attract professional phagocytes toward the dying cell. Second, “eat-me” signals appear on the surface of the apoptotic cell, which will help phagocytes recognize and engulf it. Lastly, the internalized apoptotic body is degraded in the phagolysosomal compartment by proteases, DNAses and lipases.

During apoptosis, cellular components are modified by the activity of caspases and packaged into sealed vesicles—the so-called apoptotic bodies—that expose phosphatidylserine (PS) (9). The activation of *initiator* caspases (2, 8–10) leads to the cleavage-dependent activation of the *effector* caspases (3, 6, 7), which, being promiscuous proteases, cause the widespread cleavage of proteins in the cell (10). This, in turn, promotes the degradation of nuclear and cytoskeletal proteins and the activation of accessory enzymes, such as the caspase-activated DNAse (CAD), that degrades chromosomal DNA (11). The concomitant cleavage of nuclear scaffold proteins such as lamins leads to nuclear fragmentation (12), while proteolysis of actin, fodrin, and gelsolin (13) causes cell shrinkage and membrane blebbing. In addition, caspase activation is central to drive PS exposure on the outer leaflet of the plasma membrane, a key event in apoptotic cell recognition and clearance (14). Thus, caspase activity is largely accountable for the morphological and biochemical hallmarks of apoptosis, including the auto-digestion of cellular components and the generation of “find-me” and “eat-me” signals. Caspases can be activated when proteases that are normally secreted are released into the cytosol. For example, neutrophil elastase induces the unfolded protein response in vascular endothelial cells, promoting apoptosis via caspase-3/7 activation (15). The best-characterized apoptotic “find-me” signals are the nucleotides ATP and UTP (16), the chemokine CX3CL1 (17), ICAM3 (18), and the lipids lysophosphatidylcholine (LPC) (19) and sphingosine 1-phosphate (S1P) (20). Interestingly, these signals can be released as soluble mediators or become exposed on the surface of apoptotic microparticles, which detach from the main apoptotic body and are capable of diffusing in the extracellular environment (21).

Apoptotic bodies are easily engulfed by leukocytes (professional phagocytes) and are cleared from the tissue without any inflammatory impact. This process depends largely on the exposure of PS on the outer leaflet of the membrane, an evolutionary conserved “eat-me” signal for apoptotic cells. PS is recognized by a plethora of receptors, including TIM-1, TIM-3, TIM-4, BAI1, MerTK, and the stabilins 1 and 2, which will cause internalization of the apoptotic bodies by phagocytes (2, 22–25). Also, scavenger receptors such as CD36, might be able to interact directly with exofacial PS due to its negative charge (26). In addition to direct receptor-binding to PS, several soluble molecules were described to bridge phagocyte receptors to the phospholipid. They may originate from the phagocyte, the dying cell or the interstitial fluid. Examples of phagocyte-derived bridging molecules include milk fat globule EGF factor 8 (MFG-E8), developmental endothelial locus 1 (Del-1), growth arrest-specific 6 (Gas6), protein S and the complement factor C1q. Bridging proteins interact with PS via different PS-binding domains. For example, MFG-E8 secreted by macrophages and immature dendritic cells binds to PS on apoptotic cells via its Ca^2+^-independent discoidin-like C2 domain, while interacting with α_ν_β_3/5_ integrins on the phagocyte membrane, resulting in cell engulfment (27, 28). In contrast, Gas6 and protein S bind PS via their γ-carboxyglutamic acid (Gla) domain. Unlike the discoidin-like C2 domain, binding of the Gla domain to PS requires Ca^2+^, in this way promoting apoptotic cell internalization (29, 30). C1q binds to apoptotic cells via its cationic globular head, and interacts with calreticulin-CD91 on phagocytes to promote efferocytosis (31, 32). Another phagocyte-derived protein, Annexin A1, can be translocated to the plasma membrane to interact with PS exposed on the apoptotic cell target, and this may contribute significantly to the anti-inflammatory effects of apoptotic cell clearance (33).

Complement C1q and additional bridging molecules such as IgM and collectins were proven to bind to “defects” in the plasma membrane of the apoptotic cell, including the presence of phosphatidylcholine, phosphatidylethanolamine, lyso-phospholipids, carbohydrates, and DNA (34). Collectins, such as mannose-binding lectin (MBL) and complement C1q, bind late apoptotic cells and also drive engulfment via interaction with CD91 and calreticulin on the macrophage *in vitro*. Calreticulin is an endoplasmic reticulum (ER)-localized chaperone that normally facilitates folding and quality control of N-glycosylated proteins. As cells undergo apoptosis, calreticulin escapes and translocates to the plasma membrane, where it acts as an “eat-me” signal that is recognized by CD91 on phagocytes (35). In addition, a variety of other receptors and adaptor molecules have been reported to contribute to efferocytosis. These include Fcγ receptors, β2-glycoprotein I receptor, lectins, CD14, ABC transporters, scavenger receptors, and complement components [reviewed in (31, 36–42)]. Together this indicates that there is marked redundancy in receptors and ligands for the engulfment of apoptotic cells.

Interestingly, although PS exposure is a hallmark of apoptosis, forced PS exposure on viable cells does not trigger internalization (43). This is due to the presence of “don't eat-me” signals on viable cells, including CD31, CD46, CD47, and CD61, which disable target cell engulfment. The downregulation of “don't eat-me” signals, such as CD47 and its binding partner SIRPα, contributes to internalization of apoptotic bodies, indicating that a coordinated effort between the dying cell and the phagocyte likely exists (44).

## Non-Apoptotic Cell Death

Necrosis is generally considered to be a drastic and uncontrolled form of cell death, characterized morphologically by nuclear and organellar swelling (oncosis) and plasma membrane rupture (Figure 1) (10). Due to the loss of membrane integrity, the intracellular contents are spilled out by the dying cell. The exposure of necrotic cell content (or debris) is abrupt and lacking in processing, causing it to be released in a disorderly fashion into the tissue, without the specific cues of its apoptotic counterpart. This causes necrotic debris to be potent inducers of inflammation, through activation of pattern-recognition receptors such as toll-like receptors (TLRs), NOD-like receptors (NLRs), and C-type lectins (CLECs), among others. Generally, necrotic cell death is problematic to tissues, prompting the need for immediate removal of debris, delaying the regeneration required after injury and sustaining collateral inflammatory damage. Interestingly, the recent identification of signaling pathways that are activated before and during necrosis have prompted reconsideration of this type of cell death as multiple, distinct types of events, at least some of which are tightly regulated and not always accidental. Recent subclassifications of necrosis include pyroptosis, necroptosis, parthanatos, ferroptosis, oxytosis, ETosis, and secondary necrosis (45).

**Pyroptosis** is a type of necrotic cell death caused by extensive inflammasome activation. It occurs in cell types that express inflammasome components, such as macrophages, upon infection (e.g., with the intracellular pathogen *Salmonella typhimurium*) or LPS treatment (46). Pyroptosis is caused by the formation of gasdermin D pores, which assemble at the plasma membrane after proteolytic processing of their precursor by inflammasomes containing activated caspase-1 or -11 (47, 48). Gasdermin pores create a path for the release of IL-1β, but secondarily cause cell lysis by excessive permeabilization of the plasma membrane. **Necroptosis** differs from other modalities of necrosis by the involvement of receptor-interacting protein kinase 1 (RIPK1) and RIPK3, which recruit and phosphorylate the mixed lineage kinase domain-like protein MLKL (45). Subsequently, MLKL oligomerizes, translocates to the inner leaflet of the plasma membrane and promotes membrane permeabilization and cell death (49, 50). Curiously, necroptosis requires the inhibition of caspase-8, which otherwise causes the cells to die by apoptosis. This may restrict the relevance of this death pathway *in vivo*, since caspase-8 inhibition may only occur in some viral infections (51). **Parthanatos** is a necrotic mode of cell death that depends on poly(ADP-ribose) polymerase proteins (PARPs). PARPs are typically activated by DNA breaks from ultraviolet light and by alkylating agents (52). By causing poly (ADP-ribosyl)ation of target proteins, PARPs may deplete cells of NAD^+^ and consequently of ATP, causing necrotic cell death.

**Ferroptosis** is the necrotic cell death induced by iron-dependent oxidative stress (53). It was postulated that iron catalyzes the lipid peroxidation triggered by the ferroptosis-inducing molecules erastin and RSL3, or by inhibiting the glutamate/cystine antiporter. It was later found that these pathways converge on the reduction of intracellular glutathione (GSH) levels and impaired GSH peroxidase 4 (GPX4) activity, leading to the accumulation of lipid-based reactive oxygen species and cell death (54). As expected, iron-chelators and lipophilic antioxidants were found to be potent inhibitors of ferroptosis (55). A related oxidative stress-dependent necrotic cell death, **oxytosis**, involves GSH depletion, 12-lipoxygenase activation and opening of cGMP-gated channels on the plasma membrane (56). This leads to calcium influx and activation of the calpain-cathepsin cascade, causing lysosome membrane permeabilization and necrosis (57).

In contrast to the “passive” nature of classical necrosis, one of the necrotic death pathways involves purposeful intracellular content exposure. Since its description in 2004 by Zychlinsky and collaborators (58) (neutrophil) extracellular traps (ETs), which consist primarily of extruded DNA, have been studied extensively. Subsequent work determined that cells may die during ET production, a process dubbed NETosis (59, 60). NETosis was later shown to occur in several cell types other than neutrophils, such as monocytes (61), mast cells (62), and eosinophils (63), making the name ETosis more appropriate. Generally, ETosis requires NADPH oxidase-dependent reactive oxygen species production, leading to chromatin decondensation, nuclear disruption and release of chromatin complexed with granular/cytoplasmic proteins (59), although the mechanisms underlying the process may vary between cell types.

Necrosis may also take place even after apoptosis has occurred. If apoptotic cells are not cleared in a timely fashion, the apoptotic bodies may decay and lose plasma membrane integrity, leaking their contents in a similar manner as a primary necrotic cell would (64). This event is named secondary necrosis and it shares common features with both apoptotic and necrotic cell death. The intracellular debris produced by secondary necrosis undergo apoptotic caspase processing, causing it to be qualitatively different from primary necrotic debris (65). For instance, secondary necrotic debris are considerably smaller, contain digested chromatin, prostaglandin E2 and high levels of uric acid, but very low ATP levels (65). These change drastically the manner by which the organism deals with the debris, as exemplified by the higher efficiency of complement C1q and DNAse I in degrading chromatin from secondary necrotic cells (66) and the potent anti-inflammatory polarization of macrophages elicited by C1q-covered late apoptotic debris (67).

## Necrotic “Find-me” Signals and Their Receptors

In contrast to apoptotic “find-me” signals, necrotic cells may not have enough time or energy to process their own signals. A myriad of molecules has been shown to be released by dying cells, many of which fall into the category of damage-associated molecular patterns (DAMPs): *bona fide* cellular components that are normally concealed inside the cell, but that become exposed to the extracellular environment upon cell damage or death. Some well-established DAMPs include mitochondria-derived N-formylated peptides, DNA and RNA, the nuclear protein HMGB1, histones, actin, calcium-binding S100 proteins, heat-shock proteins (HSPs), ATP and uric acid, among many others (68, 69). Necrotic cells may also release pre-stored inflammatory mediators, such as IL-1α, IL-33, and chemokines, which may directly or indirectly recruit phagocytes to the vicinity. In addition, the occurrence of necrosis and the consequent exposure of “unusual” molecules promptly activates the proteolytic cascades of complement and coagulation. The activation of complement on necrotic debris can itself generate several “find-me” signals, including the powerful chemoattractant C5a. Below, we discuss established necrotic “find-me” signals. After reading this section the reader may agree that neutrophils and monocytes respond to a “complex pool of exogenous signals, of which no single cue is absolutely required for migration” (70).

### Formyl-Peptides

Formyl-peptides (or *N*-formylated peptides) are classically generated in the course of bacterial protein synthesis, which is initiated by *N*-formyl-methionine residues. Mitochondria, sharing the bacterial ancestry, initiate protein synthesis similarly, thus creating a formylated protein reservoir inside the eukaryotic cell. Upon necrosis, the release and cleavage of mitochondrial proteins produces formyl-peptides, causing massive leukocyte activation and recruitment in a variety of necrotic states (71, 72).

Formyl-peptides are powerful chemoattractants. The most used analog, fMLP, activates neutrophils in the picomolar range (73, 74). It is considered an end-target chemoattractant, which bypasses the signaling of intermediate chemotactic molecules such as CXCL8 (IL-8) and LTB4 (75). Formyl-peptides bind FPR1, FPR2, and FPR3 receptors, although the classical chemotactic effects are mainly mediated by FPR1 activation. Whereas, FPR1 and FPR2 are expressed in several cell types, especially neutrophils and macrophages, FPR3 is much less understood (76). Upon ligand binding, FPR1, induces multiple intracellular signaling pathways: Gα activation signals via the MAPK pathway and the small GTPases CDC42 and RAC to stimulate migration and phagocytosis; Gβγ transduce signals via PI3Kγ and PLCβ to stimulate superoxide production and transcriptional regulation in phagocytes (77).

The role of formyl-peptides as a necrotic “find-me” signal is firmly established in the literature. In a seminal paper where McDonald et al. (71) used focal thermal injury of the liver, FPR1 activation of neutrophils was the key step required for migration into the necrotic area. The neutrophils initially traveled to the liver stimulated by an intravascular gradient of CXC chemokines. Upon reaching the edge of the necrotic site, the neutrophils switched to a FPR1-dependent migration mode, presumably chasing formyl-peptides emanating from the mitochondria of necrotic cells. Furthermore, the dependence of neutrophils on formyl-peptide gradients for recruitment to necrotic sites was confirmed in clinically-relevant disease models of drug-induced liver injury (78) and liver ischemia-reperfusion (79). Interestingly, when the necrotic injury is extensive enough, as in severe trauma (crushes, fractures, burns) or acute liver failure, the formyl peptides can be released in such a significant amount that they cause systemic inflammation, affecting lung function in mice and humans (72, 78).

Though most studies have focused on neutrophil chemotaxis and activation by formyl-peptides, macrophages also express FPR1 and are sensitive to formyl-peptide stimulation. Human PBMCs produce significant amounts of CXCL8 in the presence of mitochondrial extracts containing formyl-peptides (80). Interestingly, the response is stronger when formyl-peptides are applied in conjunction with other stimulatory DAMPs such as HMGB1. This suggests that formyl-peptides may indirectly recruit phagocytes to necrotic sites by inducing the production of additional chemoattractants (CXCL8) by resident macrophages. Formyl-peptides induce monocyte recruitment *in vitro* (81, 82), however, the relevance of FRP1 signaling in monocyte recruitment to necrotic sites *in vivo* remains elusive.

Importantly, questions about formyl-peptides as necrotic “find-me” signals remain. For example, the mechanism by which peptides are retained in necrotic areas in order to signal to leukocytes is unclear. Chemokines interact with glycosaminoglycans in order to form a gradient in the vasculature (83), but no mechanism has been proposed for the gradient formation of formyl-peptides. One should also consider that necrotic formyl-peptides may be very heterogeneous, varying in peptide length from a few to several amino acids. This may also impact the localization and agonistic activity of the formyl-peptides *in vivo*.

### Chemokines

Chemokines are chemotactic cytokines that dictate the localization and mobilization of leukocyte populations in the organism (84). Chemokines are produced in a constitutive fashion and/or in response to stimuli such as those that activate pattern-recognition receptor (85). In the context of necrosis, chemokines play the roles of primary and secondary “find-me” signals, meaning that they can originate from both the dying cells and from healthy bystander cells. However, the multitude and promiscuity of chemokines and chemokine receptors adds a significant layer of complexity to the study of these mediators *in vivo* (84). CC and CXC chemokines can be released essentially by any cell type, including resident leukocytes (85). For instance, the chemokine CXCL1 can be produced by endothelial cells, pericytes, hepatocytes, macrophages, and fibroblasts (86–89). In addition, leukocytes can produce chemokines in an autocrine fashion, such as when neutrophils secrete CXCL2 during transendothelial migration (87) and Kupffer cells that release CCL2 during necrotic injury (90). Thus, there is an abundance of chemokine sources that can direct the migration of phagocytes to necrotic sites, reflecting the importance of chemokines as necrotic “find-me” signals.

The chemokines CXCL1 and CXCL2 (in mice) or CXCL8 (in humans), among others, have been known as powerful chemoattractants for neutrophils for decades (91). They activate CXCR1 and CXCR2 receptors to induce neutrophil polarization and migration, an effect strongly dependent on PI3Kγ signaling (92). CXC chemokines were shown to be the first signal guiding neutrophils to sites of focal necrosis in the liver (71) and suffice to induce neutrophil accumulation in zebrafish *in vivo* (93, 94). In contrast to formyl-peptides, the CXC chemokines were actually shown to form an intravascular gradient in the vicinity of necrotic areas, which is required for proper neutrophil recruitment to the injury site. These chemokine gradients are built on heparan sulfate proteoglycans expressed by the endothelium; they are long lasting and extend hundreds of microns from the site of injury (71, 94). This promotes the recruitment of patrolling neutrophils from the vasculature far from the original insult area. Moreover, CXCL1/CXCL2 signaling via CXCR1/CXCR2 receptors act in conjunction with formyl-peptides in the case of widespread hepatic necrosis (e.g., drug-induced liver injury), in which both pathways are required for maximal neutrophil recruitment to the interior of necrotic areas (95). Despite being considered redundant, evidence shows that CXCL1 and CXCL2 act on neutrophils sequentially to promote successful diapedesis and recruitment to inflamed muscle (87). Conversely, there is mounting evidence that neutrophils expressing lower levels of CXCR1 may transmigrate in reverse into the bloodstream (96). This has been corroborated by observations of neutrophil reverse migration (e.g., away from the site of necrosis and back into the bloodstream) in both zebrafish (97) and mice (98). This suggests that the role of CXC chemokines as “find-me” signals may be much more complex than anticipated, regulating initially the recruitment of neutrophils to necrotic areas and subsequently directing their egress back to the vasculature.

CC chemokines such as CCL2, CCL3, and CCL5 are notably active in cells of the monocytic lineage. Even though they are present in a variety of parenchymal and non-parenchymal cells, the CC chemokine receptors, especially CCR2, are highly expressed in monocytes and macrophages (85). Using transgenic mice, Dal-Secco et al. identified two different monocyte subsets that are recruited to necrotic sites: a classical pro-inflammatory CCR2hi-CX3CR1low population and an alternative patrolling CCR2low-CX3CR1hi population (99). They showed that CCR2hi monocytes migrate to the edge of the necrotic area after the initial wave of neutrophil recruitment, and this was dependent on CCR2 expression. The monocytes persisted in the necrotic area for days, where they transitioned *in situ* into a CX3CR1hi population. The reprogramming of the monocyte population was dependent at least partially on the cytokines IL-4 and IL-10, and was required for the timely resolution of the necrotic injury. Interestingly, CCL2 and CCL3 were found to be significantly increased in the necrotic liver of humans, correlating to a CCR2-dependent recruitment of CD68-positive monocytes to the necrotic areas (100).

Of note, chemokine receptors other than the ones mentioned above may control different leukocyte populations, playing roles that are still unclear in the context of necrotic debris. CXCR3 and its ligands CXCL9 and CXCL10 seem to control the population of NK and NKT cells, such that deficiency of CXCR3 causes a significant reduction of both cell populations in necrotic liver (101). Similarly, the chemokine CXCL12, known to control neutrophil egress from the bone marrow via CXCR4 (102) may control later events in tissue necrosis, such as re-vascularization (103).

### Leukotriene B4 (LTB4)

LTB4 is a mediator derived from membrane phospholipids. The activation of cytosolic phospholipase 2 (cPLA2) initiates the cleavage of phospholipids to generate arachidonic acid. This fatty acid is used as substrate by the lipoxygenase 5-LOX to produce LTB4, among other intermediate eicosanoids. LTB4 is a powerful chemoattractant to neutrophils. It activates the BLT1/ LTB4R1 receptor, which, coupled to Gαi, stimulates neutrophil migration via Src-family kinases and Rho GTPases (104). Since LTB4 synthesis demands several enzymatic steps, it is unlikely to be released by necrotic cells as a DAMP. Instead, it can be produced in a matter of minutes by leukocytes such as neutrophils, macrophages, and mast cells upon demand (104). However, it has been recently demonstrated that the enzymatic machinery for LTB4 synthesis can be localized to multivesicular bodies and secreted as exosomes *in vitro* (105). In this way, LTB4 may also be produced independently of the cell and travel in the aqueous environment concealed in exosomes, increasing its diffusion range and persistence in the tissue.

LTB4 is considered an “intermediate target” chemoattractant. Nevertheless, it is required for the rapid migration and concentration of neutrophils in focal necrotic sites, a phenomenon dubbed “neutrophil swarming” (106). LTB4 is produced by neutrophils recruited to necrotic foci in order to further amplify neutrophil recruitment to the area, forming the typical densely-populated clusters that are associated to neutrophil swarming. Neutrophil-derived LTB4 can act as a signal relay molecule (107) that is necessary for cell-cell communication to produce optimal aggregation of neutrophils at the injury site (106). Moreover, LTB4 was shown to act in conjunction with other necrotic “find-me” signals such as formyl-peptides and chemokines (106, 107), supporting the idea that there is no absolute necrotic “find-me” signal, but instead, a synergistic pool of signals that vary in chemotactic potency and range to mediate an integrated response.

The importance of LTB4 as a necrotic “find-me” signal has been confirmed in several models other than laser-induced focal skin injury. In spinal cord injury, LTB4-BLT1 signaling was required for the recruitment of neutrophils to the injury site (108). Interestingly, BLT1 knockout or pharmacological blockage of the receptor reduced neutrophil recruitment significantly, but did not alter monocyte recruitment to the injured spinal cord area. In the K/BxN model of inflammatory arthritis, inhibition of 5-LO led to a significant reduction of neutrophil migration to arthritic joints and amelioration of the disease (109). There, LTB4 was also produced locally by infiltrating neutrophils. In drug-induced liver injury, deficiency of 5-LO prevented mortality associated with acetaminophen overdose, which was correlated with reduced recruitment of phagocytes to the necrotic liver (110).

It is clear that LTB4 is an essential necrotic “find-me” signal to neutrophils. Yet, many questions pertaining its production and release still remain. For example, the transport of lipid mediators across membranes is still poorly defined, as is its mode of release from the nanoscopic exosomes. It would be interesting to assess whether exosomes are able to bind to the vasculature, perhaps stimulating leukocyte recruitment at long range. In addition, based on the role of LTB4 in the skin, one could wonder if it is especially relevant in tissues with abundant extracellular matrix, where it would be better retained and less prone to degradation.

### Hydrogen Peroxide (H_2_O_2_)

H_2_O_2_ is a reactive oxygen species commonly generated in organelles such as mitochondria and phagosomes (111). The signaling capabilities of H_2_O_2_ are not limited to mammalian cells: it also serves as a major chemotactic signal in other species, such as zebrafish (*Danio rerio*) and *Drosophila*. Niethammer et al. showed formation of a H_2_O_2_ gradient minutes after wounding zebrafish, which extended up to 200 μm from the site of injury (112). The H_2_O_2_ was created by the activity of the enzyme Duox, a NADPH oxidase expressed in epithelial cells, and was necessary for rapid leukocyte recruitment to the injury site. In *Drosophila*, hemocytes also respond to H_2_O_2_ emanating from the wound (113). In this species, H_2_O_2_ was also derived from Duox and inhibition of the enzyme by siRNA knockdown or using diphenylene iodonium blocked the recruitment of hemocytes to the wound. Interestingly, it was later shown that wounding of tissues in zebrafish and flies causes a calcium wave across the tissue, which precedes and is responsible for the activation of Duox via its EF-hand motif, initiating the production of the H_2_O_2_ gradient (114).

Leukocytes must have a mechanism to sense this transient H_2_O_2_ gradient emanating from the injured cells. The redox sensor is seemingly the Src family kinase Lyn, which is activated by wound-derived H_2_O_2_ and mediates recruitment of neutrophils to injury sites in zebrafish (115). Oxidation of cysteine C466 by H_2_O_2_ activates Lyn, which in turn contributes to neutrophil migration toward the wound. Of interest, H_2_O_2_ is also chemotactic in murine (116) and human neutrophils (115), and Lyn is expressed in all mammalian leukocytes, with the exception of T cells (which nevertheless express related Src-family kinases). Thus, the role of H_2_O_2_ as a necrotic “find-me” signal spans several species and leukocyte types. Beyond the direct effects that H_2_O_2_ has on phagocyte recruitment to injury sites, it can also regulate the chemotactic responses to other “find-me” signals, such as fMLP, LTB4, and CXCL8 (117). Indeed, generation of reactive oxygen species by the NADPH oxidase at the leading edge of neutrophils is important to oxidize and inhibit the phosphoinositide phosphatase PTEN, maintaining high levels of PI(3,4,5)P_3_ at the leading edge and supporting the directional migration of neutrophils (118).

### Purines

Nucleotides are among the earliest molecules released by damaged and dying cells (119). Nucleotide sensing occurs via P2Y and P2X receptor families, which are G protein-coupled receptors and nucleotide-gated ion channels, respectively. These receptors are numerous and vary in sensitivity to different nucleotides (e.g., ATP, ADP, UTP), but the majority of the studies have focused on the role of ATP and its degradation products. ATP is very abundant in the cytoplasm, ranging from 3 to 10 mM (120). When released actively or passively by cells, ATP is rapidly hydrolyzed by ectonucleotidases into ADP, AMP, and adenosine (119). Yet, despite its very short half-life outside the cell, ATP nevertheless has pivotal effects in leukocyte activation and migration.

Chen and collaborators demonstrated that neutrophils exposed to a gradient of fMLP release ATP at the leading edge of the cell, amplifying the chemotactic response to the formyl-peptide. This effect was mediated by ATP signaling via P2Y2 receptors and subsequently by activation of A3 receptors by adenosine derived from ATP hydrolysis (121). P2Y2 activation by ATP was also required for chemotaxis of human neutrophils toward CXCL8 (122), but in this case adenosine signaling did not play a role. Moreover, it was demonstrated that macrophages utilize the same autocrine ATP amplification loop to migrate toward C5a. Blockage of P2Y2 also impaired macrophage chemotaxis *in vitro* and *in vivo* (123). Clearly, purinergic signaling is involved in phagocyte migration to several stimuli, but this is not sufficient to characterize it as a chemotactic agent. Indeed, it was demonstrated that ATP itself is not directly chemotactic to macrophages. Instead, it induces lamellipodial extensions and chemokinesis (increased random displacement) (124), without directing the migration. These studies suggest an indirect effect of ATP, that though not acting as a chemoattractant, acts in an autocrine capacity in phagocytes to maximize the response to other chemoattractants, including fMLP and chemokines.

ATP was initially implicated as a “find-me” signal of apoptotic cells (16). The authors showed that ATP and UTP released during apoptosis were required for monocyte migration toward supernatant of apoptotic cells, in a P2Y2-dependent manner. Also, the migration of monocytes toward apoptotic cells *in vivo* was impaired in the absence of P2Y2. In necrotic injuries, the role of purinergic signaling is even more interesting. Applying focal necrotic injury to the liver, it was shown that ATP is required for invasion of peritoneal macrophages into the necrotic area (125). Curiously, the peritoneal macrophages, which can be found floating in the peritoneal fluid, took this avascular route to the necrotic site by recognizing ATP released from the dead cells, which prompted the macrophages to arrest at that site. The use of apyrase (to degrade ATP and ADP) and P2X7 blockage reduced significantly the infiltration of macrophages from the peritoneum to the injury site. In the case of focal injury of the brain, the extension of microglial processes to the area of injury was also found to be mediated by ATP (126). The rapid convergence of microglial extensions to the necrotic site took place without displacement of the main cell body and was dependent on ATP and P2Y receptors. Uderhardt and collaborators found a similar response of peritoneal macrophages to a focal necrotic injury. Sessile resident macrophages extended membrane processes toward dead cells in order to cloak the debris from patrolling neutrophils, thereby minimizing inflammation (127). The extension of the macrophage processes could be blocked by apyrase or joint inhibition of P2X and P2Y receptors, indicating an elevated redundancy in purinergic signaling. In zebrafish, wounding causes ATP release and P2Y receptor activation, which in turn activates Duox to produce H_2_O_2_, recruiting phagocytes to the injury site (128). In this species, the effects of the nucleotide are not limited to phagocytes, as ATP is also involved in rapid wound closure by stimulating epithelial cell motility (129).

It is important to highlight the differences in the function of P2Y and P2X receptors. As mentioned, P2Y receptors, especially P2Y2, have been implicated in regulating the migration of phagocytes to diverse necrotic “find-me” stimuli. P2Y receptors are metabotropic, transducing signals via RhoA, Rac and PLCβ, leading to cytoskeletal rearrangement and increased intracellular calcium (130). P2X receptors, on the other hand, being ion channels activated by nucleotides, signal in a fundamentally different way. A classic example is the role of P2X7 in inflammasome activation in macrophages. In this instance, P2X7 mediates K^+^ efflux from cells stimulated by ATP, a major step in the activation of the inflammasome complex and caspase-1 that eventually culminates in the release of interleukin-1β (IL-1β). IL-1β is able to prime the production of several other chemotactic agents such as chemokines and lipid mediators, but like P2X7, lacks intrinsic chemotactic activity.

### Complement

The complement system comprises an evolutionary ancient set of fluid-phase proteins and receptors, present in vertebrates and invertebrates. It is at the core of the immune system and mediates a cross-talk between innate and adaptive responses (131–134). The complement cascade is activated by a myriad of self and non-self molecules, which initiate the proteolytic cleavage of complement proteins into fragments that deposit onto the target or are released into the extracellular fluid to signal to neighboring cells and leukocytes.

Necrotic debris can initiate complement activation through all 3 pathways: classical, alternative and lectin (133). Exposure of intracellular components such as DNA or mitochondria activate complement directly (135, 136), and natural IgM and IgG autoantibodies can bind necrotic debris to initiate complement by the classical pathway (137). In addition, there are numerous adaptors and pattern-recognition receptors that detect necrotic debris and initiate the complement cascade by themselves, including mannose-binding lectin (MBL), pentraxins, ficolins, and histidine-rich glycoprotein (34). In the specific context of this section, the production of complement C3a and C5a fragments (anaphylatoxins) is central, since these stimulate chemotaxis of leukocytes (138). Their respective receptors, C3aR and C5aR, that are expressed primarily in myeloid cells, are G protein-coupled receptors signaling via PI3K activation, MAPK activation and intracellular calcium mobilization (139).

The activation of complement on dying or necrotic cells, measured by the deposition of C3b/iC3b, has been demonstrated in several tissues, including the liver (140–142), muscle (143, 144), brain (145, 146), joint (147, 148), and intestines (149, 150). The presence of complement deposited on damaged tissues was already strong indication that C3a and C5a were being generated, but subsequent studies focused on the exact role of each fragment in disease. In muscle injury induced by cardiotoxin, it was shown that complement is activated via the alternative pathway (spontaneously), and that C3a-C3aR signaling was required for monocyte migration to the tissue (143). Deficiency in C3aR reduced the recruitment of monocytes to the injured muscle significantly, although neutrophil migration was unaffected. Moreover, C5aR was not required for the migration of either monocytes or neutrophils to the muscle, indicating specificity of C3a activity in this setting. In liver injury by ischemia/reperfusion, neutrophil migration requires complement activity. C5a is produced early during injury and formation of the complement membrane attack complex (MAC) plays an additional role in amplifying neutrophil recruitment, likely via release of IL-1β and additional DAMPs (140).

Complement inhibition in intestinal ischemia/reperfusion injury, a severe model of intestinal damage, also minimizes neutrophil recruitment and disease severity (149, 150). Interestingly, whilst complement inhibition presumably inhibited the generation of C3a and C5a in the injured intestine, it also inhibited the production of another chemoattractant, LTB4. This shows again a synergistic relationship between different classes of “find-me” signals, acting simultaneously or sequentially to guide leukocyte recruitment to necrotic debris. In the joints, the synovium is a site of both synthesis and deposition of complement (131). The alternative complement pathway plays a major role in the pathogenesis of arthritis from the initiation phase (when synoviocytes can be damaged directly by complement) to the chronic inflammatory stage (147, 148). Importantly, both C3aR and C5aR are required for the recruitment of neutrophils and macrophages to the damaged joint (148), showing yet again a degree of redundancy in the role of anaphylatoxins in phagocyte recruitment to the joint. Altogether, there is abundant evidence that complement by-products are released during necrosis and that they play a role in attracting phagocytes to injury sites.

## Necrotic “eat-me” Signals and Their Receptors

**Figure 2:**
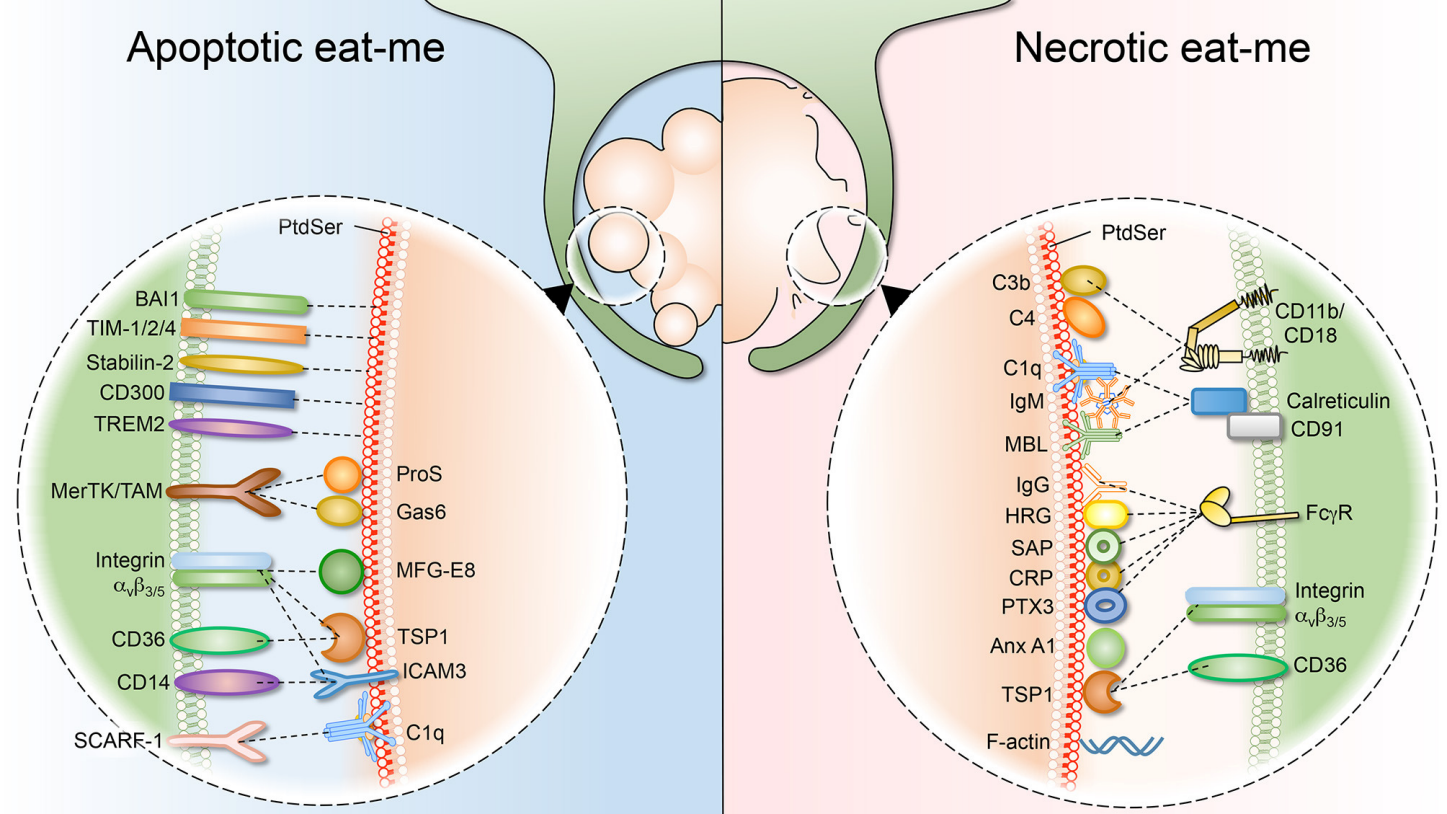
Apoptotic and necrotic “eat-me” signals, and their respective phagocytic receptors. **(Left)** As cells undergo apoptosis, they expose “eat-me” signals on their surface. The best studied eat-me signals for apoptotic cells is phosphatidylserine (PS). PS can either be bound directly by macrophage receptors such as BAI1, TIM 1/2/4, Stabilin-2, CD300, and TREM2. Alternatively, bridging molecules such as ProS, Gas6, MFG-E8, TSP1, and ICAM function to connect macrophage receptors (MerTK/TAM, integrin a_v_β_3/5_, CD36, and CD14) to the apoptotic surface. **(Right)** Necrotic cells also expose “eat-me” signals on their surface to engage professional phagocytes. Necrotic cells share some exposed “eat-me” signals, such as PS, with apoptotic cells, although the means of exposure likely differ. Other “eat-me” signals are unique to necrotic cells, such as deposition of C1q, MBL (Mannose-binding lectin), C3b, and C4 as well as IgG/IgM opsonization, and the subsequent involvement of integrin CD11b/CD18 and Fcγ receptors.

Apoptotic cells have to be cleared quickly and efficiently to prevent secondary necrosis, which would lead to the release of intracellular components and inflammation. Similarly, cells dying from primary necrosis need to be removed efficiently, as they could be a detrimental source of autoantigens and may trigger excessive inflammation (Figure 2). In the case of necrotic debris, the mechanism of recognition by professional phagocytes is not fully understood. As the necrotic cell can be disintegrated into small debris, it has been suggested that engulfment of necrotic cells resembles macropinocytosis, in which macrophages develop membrane ruffles which protrude around the target material (151–153). As reviewed briefly above, multiple receptors implicated in the clearance of apoptotic debris have been described. By contrast, much less is known about the receptors and ligands involved in the uptake of necrotic debris. Remarkably, as the evidence emerges, it is becoming apparent that some necrotic “eat-me” ligands overlap with the equivalent apoptotic signals. For example, necrotic cells also expose PS, although the mechanism underlying such exposure differs drastically. In line with this, many of the molecules that bridge PS for efferocytosis (complement, collectins and pentraxins) have also been shown to bind necrotic cells. Nevertheless, there are “eat-me” signals that apply uniquely to necrotic debris. For instance, complement C1q deposition represents a hallmark of necrotic debris, but it is absent on apoptotic debris (154). Another distinguishing “eat-me” signal is annexin A1, which is translocated to the plasma membrane of necrotic cells to promote phagocytic uptake (155). Below, we will summarize and discuss necrotic “eat-me” signals, comparing and contrasting them to apoptotic “eat-me” signals.

### Complement

Opsonization of targets by complement components C1q, C3b, and C4 alerts phagocytes bearing complement receptors such as CR1, CR3, and CR4. Moreover, it is clear that a functioning complement system is required for efficient handling of dying and dead cells. Recent evidence points to a role of complement deposition in the clearance of late apoptotic/necrotic cells, rather than early apoptotic cells (154). In fact, most apoptotic cells are cleared while at an early apoptosis stage, when complement plays a minor role. It is only when apoptotic cells persist into late apoptotic/secondary necrotic stages that complement opsonization enhances recognition by phagocytes (156).

Phagocytosis of late apoptotic/necrotic Jurkat cells is impaired in individuals with deficiencies in C1q, C2, C3, or C4. In contrast, the MBL and alternative pathway did not participate in phagocytosis of debris, suggesting that opsonization by C3 fragments and the involvement of the classical pathway are mostly responsible for the clearance of necrotic cells (157). Similarly, complement components C3 and C4 bind immediately to necrotic peripheral blood lymphocytes. In contrast, irradiated lymphocytes undergoing apoptosis only displayed a weak binding of complement components for up to 2 days. At day 3, when secondary necrosis had ensued, C1q, C3b, and C4 all bound with higher affinity (154). Also, the clearance of necrotic cells is increased in presence of serum, and adding C1q to C1q-depleted serum markedly increased uptake of primary necrotic cells (158). Another study investigated complement deposition on viable, early apoptotic and late apoptotic (secondary necrotic) Jurkat cells (159). In this study, binding of C3 and C4 to early apoptotic cells was similar to that of viable cells, while secondary necrotic cells had a substantial binding of C3, C4, and at some extent C1q. The necrotic cells also bound IgM, and depletion of plasma IgM abolished most of the complement binding, supporting a role for the classical pathway of complement activation on late apoptotic/necrotic cell clearance (159).

Macrophages were shown to engulf apoptotic cells after C1q and MBL opsonization. Calreticulin released from dying cells bound macrophages via CD91/LDL receptor related protein 1, and was shown to recognize the collagen tails of C1q and MBL attached to the surface of the dying cell (31). Besides IgM, C1q binding to necrotic cells can be initiated via molecules of the acute-phase protein pentraxin family. The classical (short) pentraxins—including serum amyloid protein (SAP) and C-reactive protein (CRP)—are produced in the liver in response to IL-6 and play a role as opsonins by binding to cellular debris and late apoptotic cells (160, 161). The long pentraxin PTX3 is produced by hematopoietic and stromal cells as a response to a primary pro-inflammatory signal such as LPS, IL-1β, and TNF-α. PTX3 has multiple functions including complement activation on necrotic cells that results in cell clearance and reduced tissue damage (162). However, PTX3 can also limit excessive complement activation by promoting deposition of complement Factor H, a major inhibitor of the alternative pathway of the complement system. Under normal conditions, Factor H binds to self-surfaces, where it inactivates accidental C3b deposition on healthy cells. By directing Factor H to the surface of dying cells, PTX3 limits tissue damage while still increasing phagocytic clearance (163). Moreover, both human and mouse pentraxins recognize FcγRI and FcγRII, and binding of pentraxins to cellular surfaces results in phagocyte activation (164). In line with data suggesting complement deposition on late apoptotic/necrotic cells, the collectin MBL was found to bind to both late apoptotic and necrotic cells, but not early apoptotic cells. MBL binding initiated C4 deposition onto the necrotic cells and addition of C1q inhibited MBL opsonization of cells (165). Taken together, these observations imply that complement deposition and recognition function as a mechanism for the clearance of necrotic cells and as a backup for clearance of late apoptotic material undergoing secondary necrosis.

### Phosphatidylserine

Although PS was long thought to be an exclusive marker of apoptosis, evidence is gathering that exposure of PS is also a hallmark of necrosis. In a study comparing internalization of apoptotic and necrotic cells, macrophages were shown to selectively engulf apoptotic and necrotic cells, while leaving living cells untouched. The engulfment of both apoptotic and necrotic cells was PS-dependent, suggesting that externalization of PS is a common trigger for the clearance of both types of cell debris (152). Annexin A5 (or Annexin V), commonly used as a marker of apoptosis due to its PS binding capacity, also binds to necrotic cells, supporting the occurrence of PS exposure during necrosis. Moreover, treatment with recombinant Annexin V inhibited phagocytosis of both apoptotic and necrotic cells by mouse macrophages, suggesting that PS exposure is required in both instances (152).

The difference in morphology between apoptotic and necrotic cells suggests that the mechanisms of PS exposure differ. PS exposure was observed in necrotic neurons of the nematode *Caenorhabditis elegans*, where it was facilitated by the homolog of the calcium-dependent scramblase TMEM16F and by CED-7, a member of the ATP-binding cassette (ABC) transporter family. However, rupture of the necrotic cells into particles can also readily account for exposure of PS without invoking specific externalization mechanisms. Zargarian et al. demonstrated that necroptotic cells also expose PS as an “eat-me” signal as phosphorylated MLKL translocates to the plasma membrane. The externalization of PS by necroptotic cells induced recognition and phagocytosis; they stained positive for Annexin A5 and exposed PS prior to overt permeabilization. The dying cells also released PS-exposing extracellular vesicles, thereby alerting neighboring cells of the impending cell death (166).

Although both apoptotic and necrotic cells expose PS, the efficiency of their clearance differs drastically: the engulfment of necrotic cells is considerably less effective, both quantitatively and kinetically. The mechanisms underlying this difference remain obscure, but down-regulation of “don't eat-me” signals in apoptotic, but not necrotic cells is a distinct possibility. Importantly, clearance of necrotic cells is carried out not only by phagocytes like macrophages, but also by non-professional phagocytes. In comparison to macrophages, engulfment by non-professional cells is slow and engulfment events were only detectable after 2.5 h. But, by taking up neighboring necrotic cells, non-professional cells remove a portion of the billions of cells that die daily during normal turnover (167).

### Annexin A1

Annexin A1 was first believed to translocate to the surface of apoptotic cells, where it was proposed to function as a bridging protein that facilitates their phagocytic uptake (168, 169). However, this interpretation was recently revised, as it was demonstrated that annexin A1 rarely translocates in apoptotic cells; instead, its translocation to the cell surface is rather a hallmark of secondary necrosis (155). As proposed earlier for apoptosis, in necrotic cells annexin A1 is believed to function by bridging PS to the phagocyte surface to promote uptake. This interaction also dampens the secretion of pro-inflammatory cytokines by the macrophages that ingested the necrotic cell. This implies that clearance of necrotic debris can have anti-inflammatory effects. After translocation, annexin A1 is proteolytically cleaved at the cell surface by ADAM10, which generates a small peptide with chemotactic activity toward monocytes, thus generating a monocytic “find-me” signal for the necrotic debris (170).

### Histidine-Rich Glycoprotein (HRG)

HRG is an abundant 75 kDa plasma glycoprotein that has a multi-domain structure known to interact with many ligands including Zn^2+^, heparin, heparan sulfate and plasminogen. HRG has been shown to function as an adaptor molecule that tethers plasminogen to glycosaminoglycan-bearing surfaces to regulate plasminogen activation (171). HRG was also demonstrated to distinguish between apoptotic cells and necrotic cells by binding to cytoplasmic ligands exposed by necrotic cells. This interaction, mediated by the amino-terminal domain of HRG, results in an opsonic function, encouraging the phagocytosis of the necrotic cell. In contrast, HRG does not opsonize apoptotic cells and thus, may play an important physiological role in the selective clearance of necrotic debris (34).

### CD14

Initially, it was thought that the macrophage plasmalemmal glycoprotein CD14 was specific for recognition and clearance of apoptotic cells, as treatment with an anti-CD14 antibody reduced the phagocyte interaction with apoptotic but not necrotic cells (172). CD14 also recognizes LPS and it was initially thought its interaction with apoptotic cells occurs also via its LPS-binding domain, but this view was subsequently revised (173). Indeed, unlike LPS, binding of macrophages to apoptotic cells does not generate pro-inflammatory signaling. Later studies found a significant role for CD14 also in the clearance of necrotic cells. (158). ICAM-3 on the surface of dying cells may serve as the ligand recognized by CD14 (174).

### Scavenger Receptors

Scavenger receptors were originally discovered by their capacity to recognize and remove modified lipoproteins. They are structurally diverse and recognize a variety of ligands, including DAMPs, oxidized PS and phosphatidylcholine (175). As PS is exposed on necrotic cells, this raises the possibility that PS-binding scavenger receptors may function as receptors for necrotic cells. SR-B1 and CD36 are class B scavenger receptors, and were the first cell surface receptors appreciated to recognize anionic phospholipids such as PS (26). CD36 for instance, which is highly expressed in macrophages, is involved in the phagocytosis of necrotic lymphocytes *in vitro* (158). Antibody blockage of CD36 caused a significant, yet partial, reduction in the macrophages ability to bind and internalize necrotic cells. Macrophages, dendritic cells and endothelial cells also express the scavenger receptor class F (SCARF1), that can recognize and engulf apoptotic cells via C1q (176). Given the earlier findings that C1q binds to late apoptotic/secondary necrotic cells, SCARF1 can potentially operate as a receptor for necrotic cells. In fact, loss of SCARF1 impaired uptake of dying cells, and SCARF1-deficient mice had accumulation of dying cells in tissues, leading to generation of autoantibodies to DNA-containing antigens and development of lupus-like disease (176).

## Phagocyte-Independent Clearance of Necrotic Debris

During pregnancy, a large number of multinucleated fragments of dying syncytiothrophoblasts are shed daily into the maternal circulation. These trophoblasts, shed by the placenta, are rapidly cleared from the circulation by endothelial cells during normal pregnancy in order to prevent clogging of the maternal pulmonary circulation. Indeed, failure to clear such fragments often results in pre-eclampsia. Endothelial cells can internalize dying trophoblasts regardless of whether they are apoptotic or necrotic. However, while engulfment of apoptotic trophoblasts does not induce endothelial cell activation, phagocytosis of necrotic trophoblasts causes endothelial activation and ICAM-I expression (177).

The organism also counts with acellular routes for degradation of necrotic debris. It has been shown that serum components such as the nuclease DNAse I, the complement protein C1q and the protease plasmin act in synergy to degrade chromatin even in the absence of leukocytes. For instance, the binding of C1q to necrotic chromatin strongly enhances the activity of DNAse I, even though C1q lacks nuclease activity (178). It was postulated that C1q was able to enhance the access of DNAse I to necrotic DNA, improving the degradation of debris. Moreover, plasminogen was shown to penetrate necrotic cells, where it was activated into plasmin (179). The proteolytic activity of plasmin caused the cleavage of histone H1, which in turn facilitated the cleavage of DNA by DNAse I. The synergy between these enzymes is required for the fast and effective breakdown of necrotic chromatin.

## “Don't Eat-me” Signals

Effective engulfment of dead cells entails not only the exposure of “eat-me” determinants, but requires a reduction of surface “don't eat-me” signals. Eukaryotic cells display CD47, a surface protein that is recognized by SIRPα, expressed by myeloid cells (44). CD47 functions by directly binding SIRPα on macrophages and monocytes, signaling inhibition of phagocytosis that is partly due to impaired myosin assembly at the phagocytic synapse (180).

Aging and the subsequent elimination of erythrocytes by efferocytosis correlates with a decrease in their surface CD47 (181). The importance of CD47 as a “don't eat-me” signal was demonstrated by Kojima and colleagues, who showed that dysregulation of CD47 signaling contributes to the development or atherosclerotic plaques. In this setting, instead of downregulating CD47, dying cells upregulated it, making apoptotic cells resistant to phagocytic clearance and thereby driving plaque formation. Interestingly, administration of a blocking CD47 antibody reversed this effect, stimulating efferocytosis and reducing atherosclerosis, making CD47 a potential drug target for the clinic (182).

CD47 can alter also the phagocytosis of necrotic debris. One explanation why necrotic debris are engulfed at a slower rate than apoptotic cells is that they have, comparatively, an increased surface expression of CD47. Moreover, CD47 was found to be clustered on necrotic cells, and these clusters stimulated RhoA-pMLC signaling in macrophages that promoted “nibbling” of the necrotic cells, rather than whole-cell internalization (183). This process—commonly known as trogocytosis—is shared by amoeba, lymphocytes, neutrophils and macrophages, and polarization of CD47 might explain the preferential nibbling over whole-cell engulfment.

CD46 is a widely expressed complement regulatory protein. It inhibits complement by binding C3b and C4b and acting as a cofactor for their proteolytic cleavage (184). CD46 is a “don't eat-me” signal that is lost during apoptosis and necrosis. In both types of dying cells, CD46 is clustered and shed in microparticles alongside nucleic acids and PS (185). The loss of CD46 correlates with an increase in deposition of C1q and C3b on the dying cells. However, only necrotic cells proceed to form membrane attack complexes, because they also undergo significant reduction in the expression of the complement regulators CD55 and CD59. This study indicated that the dying cells specifically lose “don't eat-me” signals that block complement activation in healthy cells, allowing them to be opsonized by complement and engulfed (185).

Several “don't eat-me” signals that have been implicated in apoptosis have not yet been investigated in the context of necrosis yet. CD31 (also known as platelet-endothelial cell adhesion molecule 1, PECAM-1) is an important “don't eat-me” signal, acting as a repulsive signal through homotypic CD31-CD31 interactions between cells. The ligation of CD31 on viable leukocytes promotes cell detachment. Apoptotic cells that lack CD31 bind tightly to leukocytes and are subsequently engulfed (186). Plasminogen activator inhibitor (PAI)-1, is a member of the serpin family of serine protease inhibitors. It appears to co-localize with calreticulin on viable neutrophils, where it is thought to impair signaling to macrophages. This impairment is lost during neutrophil apoptosis, suggesting that PAI-1 is a “don't eat-me” signal (187). Also, the urokinase receptor (uPAR), which normally plays a role in fibrinolysis, cell migration and adhesion, was shown to modulate efferocytosis (188). Macrophages from uPAR-deficient mice demonstrated enhanced ability to engulf viable neutrophils *in vitro* and *in vivo*. In line with this, expression of uPAR was reduced in apoptotic neutrophils compared to viable neutrophils, suggesting that uPAR is also a *bona fide* “don't eat-me” signal that is downregulated in apoptotic cells (189). Proteinase 3 (PR3) is a neutrophil granular protein that is co-externalized with PS during neutrophil apoptosis (190). PR3 impairs phagocytosis of apoptotic neutrophils by macrophages via inhibition of calreticulin function, a powerful “eat-me” signal. Another regulator is CD24, a heavily glycosylated GPI-anchored surface protein. It interacts with Siglec-10 in leukocytes to dampen inflammation in a variety of diseases (191). Recently, CD24 was described as a major “don't eat-me” signal exploited by tumor cells to evade the immune response (192). CD24 is overexpressed by a variety of tumor cells, inhibiting phagocytosis by neighboring tumor-associated macrophages, which express high levels of Siglec-10. Blockage of CD24 by a monoclonal antibody reduced tumor growth *in vivo*, suggesting that inhibition of this “don't eat-me” signal suffices to enable phagocytosis of live cancer cells.

## Immune Consequences of Defective debris clearance

The generation of necrotic debris is a severe occurrence; yet, it is immediately met by a barrage of fluid-phase proteins, mediators and cells, which cause it to be essentially uneventful. Tissue inflammation resolves in a timely manner and immune responses against self-develop very rarely. However, if the organism fails to contain or clear the necrotic debris appropriately, tissue inflammation is prolonged and autoimmunity can ensue. Mutations that impair the ability of leukocytes to recognize or eliminate debris have been connected to defects in tissue regeneration and to diseases such as systemic lupus erythematosus (SLE). Below, we highlight a series of studies describing the catastrophic consequences of tampering with the response to necrotic cell death.

Inhibition of phagocyte recruitment or function at necrotic sites results in a clear defect in recovery from injury. Depletion of neutrophils prevents the clearance of debris from necrotic sites, leading to an impairment of regeneration and revascularization of the focal injury (98). Moreover, inhibition of monocyte recruitment to necrotic foci, whether due to CCR2 deficiency or to interference with their transition into CX3CR1^+^ cells delays the regeneration of the necrotic injury (99). Similar observations were made in complement-deficient models, reinforcing the notion that the removal of necrotic debris by phagocytes is paramount to tissue repair.

As described above, complement contributes “find-me” and “eat-me” signals to necrotic cells, and several studies have shown its major role in tissue regeneration (132, 140, 193–195). In the long term, defects in the complement cascade have been strongly associated to the development of SLE. Although a multi-factorial disease, SLE and related lupus-like syndromes are clearly connected to mutations or deficiency in C1q, C2, C3, and C4 complement factors (196). In addition, the disease has been associated to decreased expression of complement receptors CR1 and CR2 (133). Defects in complement activation, such as in the classical pathway, also yield organisms susceptible to autoimmunity, as is the case of IgM-deficient mice (197). Interestingly, a mutation of CD11b (*ITGAM*) has been correlated to the development of SLE as well (198). Phagocytes express high levels of CD11b, which is used as both complement receptor (CR3) and as adhesion molecule (Mac-1). Whether the polymorphism affects the phagocytic or adhesive functions of CD11b is still unclear, but the findings nevertheless provide further indication that impairment of the ability of phagocytes to clear debris causes immediate and long-term disadvantages to the host.

The exposure of extracellular DNA is a key factor in SLE development. The accumulation of DNA in tissues and bloodstream has to be rapidly counteracted by the activity of DNAses to minimize inflammation and autoimmunity. An abundant source of DNAse activity in the organism is serum, which contains two major nucleases, DNAse I and DNAse IL3 (199, 200). The two enzymes have non-redundant roles in DNA/chromatin degradation; DNAse I acts preferentially against internucleosomal “naked” DNA, whereas DNAse IL3 cleaves chromatin (protein-bound DNA) with high activity (201). DNAse I deficiency causes mice to develop anti-nuclear antibodies and SLE (200). DNAse IL3 deficiency is also sufficient to cause autoimmunity and SLE in mice (202, 203). In humans, mutations of DNAse I (204), DNAse IL3 (205) and DNAse III (TREX1) (206) have already been implicated in the incidence of SLE or lupus-like disease. Global deficiency in DNAse II, an isoform found in lysosomes, is embryonically lethal due to the accumulation of undigested DNA from red cell nuclei inside macrophages, which mount a type I interferon response that leads to embryonic demise (207). Interestingly, the lethality can be abrogated by simultaneously knocking out STING, a central adaptor of cytoplasmic DNA immunity (208), suggesting that leakage of undegraded debris from the phagosome to the cytosol fuels the deleterious response. The deficiency in DNAse II also correlates with worsening of heart failure (209) and development of polyarthritis in mice (210). It is clear that removal of necrotic debris is a multi-layered response: the phagocytes must be able not only to reach and ingest the debris, but also to effectively degrade it in order to avoid overt inflammation and autoimmunity.

Like DNA, actin is a very abundant component of cells and a DAMP conserved across species (211, 212). Necrotic cells expose actin after the plasma membrane integrity is breached, and the released actin is recognized by Clec9A (DNGR-1), a C-type lectin receptor expressed primarily in dendritic cells (211, 213). Clec9A specifically recognizes filamentous (F)-actin, which—remarkably—persists in necrotic cells even after their death. F-actin is able to bind Clec9A even when forming complexes with actin-binding proteins such as spectrin, α-actinin, and myosin II (213, 214). Importantly, the recognition of actin-rich necrotic debris by dendritic cell Clec9A prompted the cross presentation of self-antigens to CD8 T cells, a mechanism that explains how autoimmunity is initiated by the exposure of necrotic debris. Exposure of F-actin can be further regulated, as it is depolymerized by circulating DNase I. Full F-actin depolymerization requires ATP, which could be present as necrotic cells release ATP (described in section Purines) (215).

## Concluding Remarks

Consideration of necrotic cells as an important, ongoing contributor to overall cell death provides a different vantage point of how debris are sensed and cleared and their contribution to inflammation and autoimmunity. Clearly, inhibiting the host's ability to eliminate and process necrotic debris has harmful effects. Strikingly, therapies for acute inflammation are largely confined to the use of anti-inflammatory drugs. In the short term, this approach reduces tissue inflammation and the associated symptoms (swelling, pain), but it comes at the cost of delayed resolution of injury. Prevention of inflammation retards debris clearance, re-growth of parenchymal cells and tissue regeneration. Thus, an alternative approach would be to stimulate debris clearance in addition to minimizing the uncomfortable symptoms. This can be accomplished by the application of pro-resolving mediators such as resolvins, lipoxins, hydrogen sulfide, IL-10, and annexin A1, which can stimulate clearance mechanisms without the damaging effects of excessive inflammation (216, 217).

## Author Contributions

JW and PM prepared the figures. JW, SG, and PM wrote the manuscript.

### Conflict of Interest

The authors declare that the research was conducted in the absence of any commercial or financial relationships that could be construed as a potential conflict of interest.
